# Adiponectin promotes VEGF-A-dependent angiogenesis in human chondrosarcoma through PI3K, Akt, mTOR, and HIF-α pathway

**DOI:** 10.18632/oncotarget.5479

**Published:** 2015-10-12

**Authors:** Hsiang-Ping Lee, Chih-Yang Lin, Jhao-Sheng Shih, Yi-Chin Fong, Shih-Wei Wang, Te-Mao Li, Chih-Hsin Tang

**Affiliations:** ^1^ Graduate Institute of Chinese Medicine, China Medical University, Taichung, Taiwan; ^2^ Department of Chinese Medicine, China Medical University Hospital, Taichung, Taiwan; ^3^ Graduate Institute of Basic Medical Science, China Medical University, Taichung, Taiwan; ^4^ Department of Sports Medicine, China Medical University, Taichung, Taiwan; ^5^ Department of Orthopedic Surgery, China Medical University Hospital, Taichung, Taiwan; ^6^ Department of Medicine, Mackay Medical College, New Taipei City, Taiwan; ^7^ School of Chinese Medicine, China Medical University, Taichung, Taiwan; ^8^ Department of Pharmacology, School of Medicine, China Medical University, Taichung, Taiwan; ^9^ Department of Biotechnology, College of Health Science, Asia University, Taichung, Taiwan

**Keywords:** adiponectin, chondrosarcoma, angiogenesis, VEGF-A, HIF

## Abstract

Chondrosarcoma is a type of highly malignant tumor with a potent capacity to invade locally and cause distant metastasis. Adiponectin is a protein hormone secreted predominantly by differentiated adipocytes. On the other hand, angiogenesis is a critical step in tumor growth and metastasis. However, the relationship of adiponectin with vascular endothelial growth factor-A (VEGF-A) expression and angiogenesis in human chondrosarcoma is mostly unknown. In this study we first demonstrated that the expression of adiponectin was correlated with tumor stage of human chondrosarcoma tissues. In addition, we also found that adiponectin increased VEGF-A expression in human chondrosarcoma cells and subsequently induced migration and tube formation in human endothelial progenitor cells (EPCs). Adiponectin promoted VEGF-A expression through adiponectin receptor (AdipoR), phosphoinositide 3 kinase (PI3K), Akt, mammalian target of rapamycin (mTOR), and hypoxia-inducible factor-1α (HIF)-1α signaling cascades. Knockdown of adiponectin decreased VEGF-A expression and also abolished chondrosarcoma conditional medium-mediated tube formation in EPCs *in vitro* as well as angiogenesis effects in the chick chorioallantoic membrane and Matrigel plug nude mice model *in vivo*. Therefore, adiponectin is crucial for tumor angiogenesis and growth, which may represent a novel target for anti-angiogenic therapy in human chondrosarcoma.

## INTRODUCTION

Chondrosarcoma are derived from the abnormal proliferation cartilage. In bone cancer, the chondrosarcoma accounts for about 26%. Usually occur at between 10 to 80 years male, and the tumor generally appears on scapula, sternum, ribs, or pelvis [1, 2]. In clinically, surgical resection remains the primary mode of therapy for chondrosarcoma. It have been reported that chondrosarcoma can easily metastasize to other organs, such as lung and liver [3]. When it occurrence of distant metastasis, the patient have a poor prognosis. Until now still lacks an effective adjuvant therapy, therefore, the development of novel molecular mechanisms is very important for chondrosarcoma metastasis [4, 5].

The tumor metastasis contains many process functions, like proliferation [6], migration [7], invasion [8], and angiogenesis [9]. Among them, the most important step is angiogenesis, because when the tumor grew to a certain size, it will require more nutrients and oxygen to supply its growth. On the other hand, the tumor metastasized to remote organs need through these new blood vessels. The vascular endothelial growth factor-A (VEGF-A) is an important protein regulating of angiogenesis [9]. It has been shown to play an important role in embryonic development, wound healing, and tumor angiogenesis [10]. In addition, many literatures also pointed out that VEGF-A is increased many tumors angiogenesis, such as breast [11], lung [12], pancreatic [13], cervical carcinoma [14], prostate [15], osteosarcoma [16], and chondrosarcoma [8]. Many drugs have been demonstrated that reducing cancer growth and metastasis through inhibited VEGF-A expression [17, 18]. Therefore, the anti-VEGF-A is a critical target for cancer therapy and metastasis.

Adiponectin (also known as Acrp30, AdipoQ and GBP28), an adipocytokine secreted by adipocytes, has been receiving a great deal of attention due to its insulin-sensitizing effects and possible therapeutic use for metabolic disorders [19, 20]. Adiponectin has emerged as a possible link between obesity and cancer. It has been suggested that decreased levels of serum adiponectin are associated with an increased risk for obesity-related cancers such as colon, breast, endometrial and prostate cancer [21–23]. Furthermore, serum adiponectin in patients with gastric cancer has been found to be inversely correlated with pathological findings such as tumor size, depth of invasion and tumor stage in patients with gastric cancer [24].

Due to the prognosis of patients with chondrosarcoma distant metastasis are very poor [25], therefore development of an anti-angiogenic and anti-metastatic therapy could conceivably be useful in these patients. Adiponectin has been reported to promote chondrosarcoma metastasis through up-regulation of integrin α2β1 [26]. However, the role of adiponectin in VEGF-A expression and angiogenesis in human chondrosarcoma are largely unknown. Here we reported that adiponectin promotes VEGF-A expression in human chondrosarcoma via activation of phosphoinositide 3 kinase (PI3K), Akt, mammalian target of rapamycin (mTOR), and hypoxia-inducible factor-1α (HIF)-1α signaling pathways. In addition, knockdown of adiponectin significantly reduced VEGF-A expression and angiogenesis *in vivo*. Our results demonstrate that adiponectin plays important role during angiogenesis in human chondrosarcoma. Therefore, adiponectin maybe a new therapeutic target for chondrosarcoma.

## RESULTS

### Adiponectin and VEGF-A expression correlates with the tumor stage of patients with chondrosarcoma

**Figure 1:**
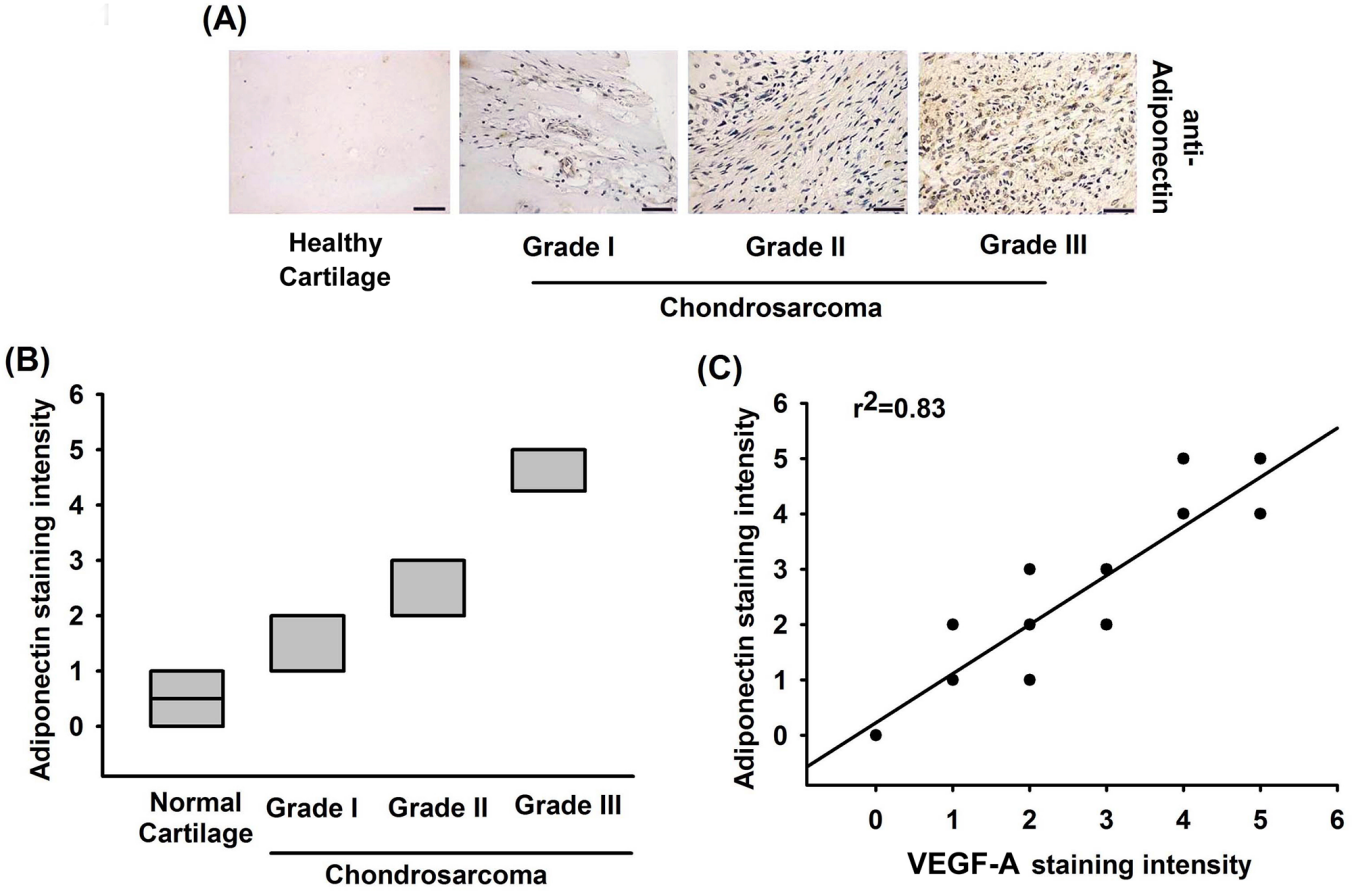
The correlation of adiponectin, VEGF-A and tumor stages in human chondrosarcoma tissues **A.** Immunohistochemistry of adiponectin expression in healthy cartilage and chondrosarcoma tissue. Scale bar = 50 μm. **B.** Quantification of adiponectin staining. The correlation data are shown in **C.** Data represent the mean ± S.E.M.

Adiponectin has been demonstrated to promote metastasis of human chondrosarcoma cells [26]. In addition, VEGF-A-promoted angiogenesis is a critical step in metastasis of chondrosarcoma [8]. However, little is known about the correlation between adiponectin and VEGF-A in human chondrosarcoma. We therefore examined human chondrosarcoma tissues for the expression of adiponectin using immunohistochemistry. The expression of adiponectin in chondrosarcoma patients was significantly higher than that in healthy cartilage (Figure 1A & 1B). We previous reported that high level expression of VEGF-A correlate strongly with tumor stage in chondrosarcoma patients [27, 28]. In addition, the high level of adiponectin expression correlated strongly with VEGF-A expression and tumor stage (Figure 1C). Overall, these results suggest that adiponectin and VEGF-A expression correlates with tumor stage in patients with chondrosarcoma.

### Adiponectin induces VEGF-A expression in human chondrosarcoma cells and subsequently promotes angiogenesis in EPCs

**Figure 2:**
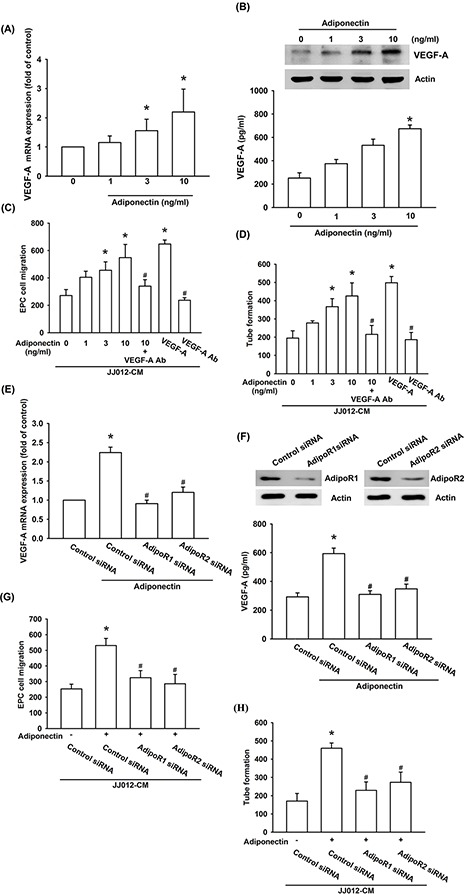
Adiponectin promotes VEGF-A expression in human chondrosarcoma cells through AdipoR1/R2 receptor **A. & B.** The JJ012 cells were incubated with adiponectin (1–10 ng/ml) for 24 h, and VEGF-A expression was examined by qPCR, ELISA, and western blotting. **C. & D.** The JJ012 cells were pre-treated for 30 min with VEGF-A antibody (5 μg/ml) followed by stimulation with adiponectin (10 ng/ml) or incubated with adiponectin (1–10 ng/ml) for 24 h. The medium was collected as CM and then applied to EPCs for 24 h. The cell migration and capillary-like structure formation in EPCs was examined by Transwell and tube formation assay. **E. & F.** The JJ012 cells were transfected with AdipoR1 or AdipoR2 siRNA for 24 h followed by stimulation with adiponectin (10 ng/ml) for 24 h, and VEGF-A expression was examined by qPCR, ELISA and western blotting. **G. & H.** In addition, the medium was collected as CM and then applied to EPCs for 24 h. The cell migration and capillary-like structure formation in EPCs was examined by Transwell and tube formation assay. Results are expressed as the mean ± S.E.M. *, *p* < 0.05 compared with control; #, *p* < 0.05 compared with adiponectin-treated group. Ab, antibody.

Next, we applied adiponectin to human chondrosarcoma cell line and determined the expression of VEGF-A. The results showed that adiponectin increased VEGF-A mRNA expression in a concentration-dependent manner (Figure 2A). We also found that adiponectin induced a concentration-dependent production of VEGF-A in human chondrosarcoma cells by ELISA assay and western blot (Figure 2B). The process of angiogenesis mainly involves endothelial cells proliferation, migration, and tube formation to form new blood vessels [29]. We then examined whether adiponectin-induced VEGF-A expression stimulated angiogenesis by using an EPCs model *in vitro*. We demonstrated that conditioned medium (CM) from adiponectin-treated chondrosarcoma cells dramatically enhanced migration and tube formation of EPCs (VEGF-A was used as positive control) (Figure 2C & 2D; Supplementary Figure S1). To elucidate adiponectin-induced VEGF-A plays an important role in angiogenesis, the VEGF-A antibody was used. Pretreatment of chondrosarcoma cells with VEGF-A antibody significantly prevented adiponectin-induced migration and tube formation of EPCs (Figure 2C & 2D; Supplementary Figure S1).

The adiponectin receptor (AdipoR)1 and AdipoR2 are associated with an invasive and metastatic phenotype of chondrosarcoma cells [26]. Therefore, we hypothesized that AdipoR may be involved in adiponectin-induced VEGF-A expression in human chondrosarcoma cells. Transfection of cells with AdipoR1 and AdipoR2 siRNA markedly inhibited adiponectin-induced VEGF-A expression (Figure 2E & 2F) and also reduced AdipoR1 and AdipoR2 expression respectively (Figure 2F upper panel). In addition, CM from chondrosarcoma cells demonstrated that AdipoR1 and AdipoR2 siRNA significantly reduced adiponectin-mediated migration and tube formation of EPCs (Figure 2G & 2H). These data suggest that adiponectin and AdipoR1/R2 interaction promotes angiogenesis by VEGF-A expression.

### The signaling pathways of PI3K, Akt, and mTOR are involved in the potentiating action of adiponectin

**Figure 3:**
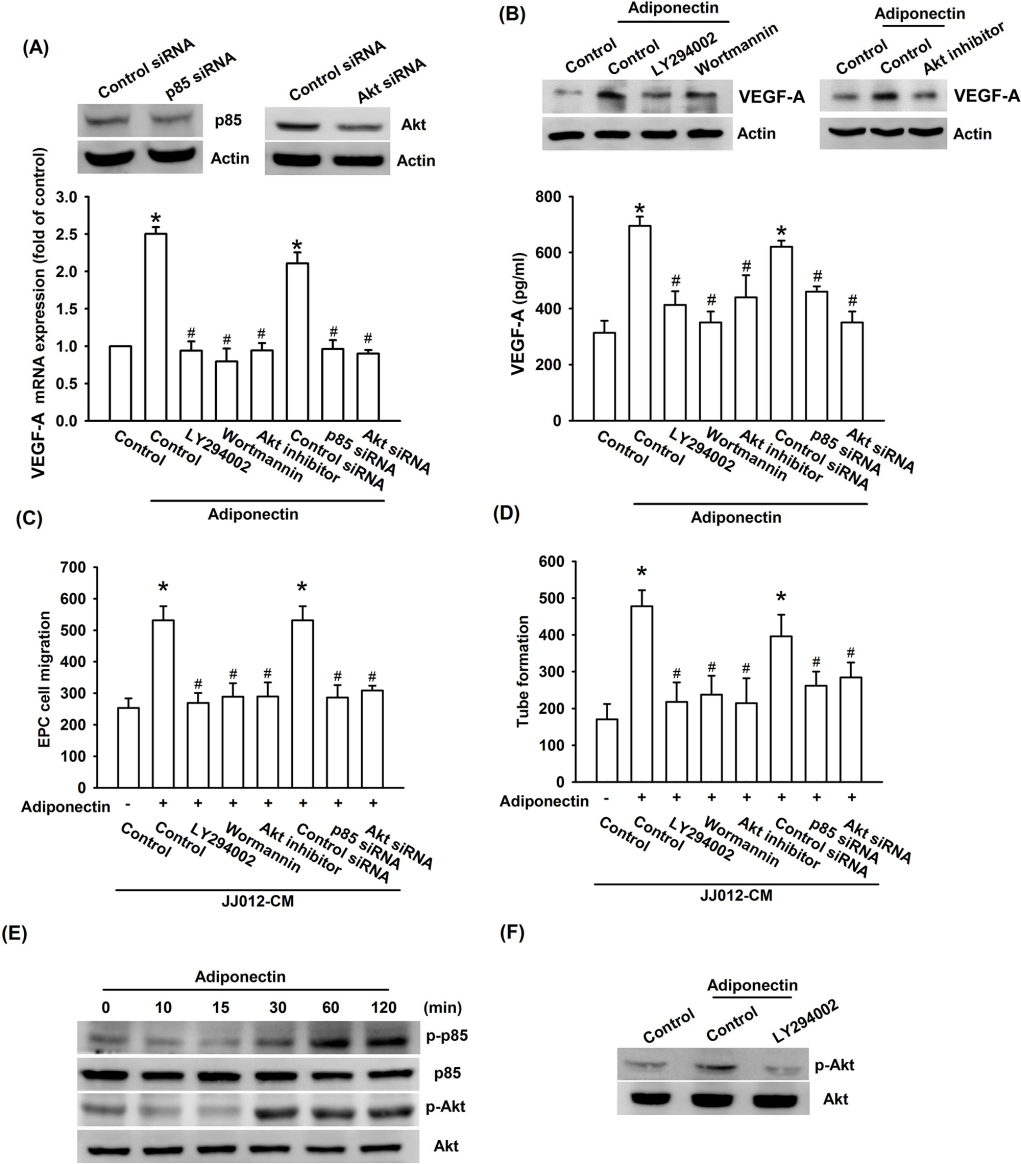
PI3K/Akt pathway is involved in adiponectin-increased VEGF-A expression **A. & B.** Cells were pretreated with the ly294002 (10 μM), wortmannin (150 nM) and Akt inhibitor (10 μM) for 30 min or transfected with p85 and Akt siRNA for 24 h followed by stimulation with adiponectin (10 ng/ml) for 24 h, and VEGF-A expression was examined by qPCR, ELISA and western blotting. **C. & D.** In addition, the medium was collected as CM and then applied to EPCs for 24 h. The capillary-like structures formation and cell migration in EPCs were examined by tube formation and Transwell assay. **E. & F.** Cells were incubated with adiponectin (10 ng/ml) for the indicated times, or pretreated with the ly294002 for 30 min followed by stimulation with adiponectin for 60 min, and the Akt phosphorylation was determined by western blotting. Results are expressed as the mean ± S.E.M. *, *p* < 0.05 compared with control; #, *p* < 0.05 compared with adiponectin-treated group.

PI3K/Akt is a common downstream signaling pathway of the AdipoR receptor [30]. We therefore examined whether PI3K/Akt pathway is involved in the adiponectin-increased VEGF-A expression and angiogenesis. The results show that pretreatment of cells for 30 min with PI3K inhibitors ly294002 or wortmannin and Akt inhibitor or transfection of cells for 24 h with p85 or Akt siRNA abolished adiponectin-induced VEGF-A expression (Figure 3A & 3B). In addition, adiponectin-increased EPC migration and tube formation was also diminished by pre-treatment with p85 and Akt inhibitor or siRNA (Figure 3A–3D). Moreover, incubation of JJ012 cells with adiponectin increased p85 and Akt phosphorylation in a time-dependent manner (Figure 3E). In contrast, pretreatment of cells with ly294002 markedly inhibited the adiponectin-induced Akt phosphorylation (Figure 3F).

**Figure 4:**
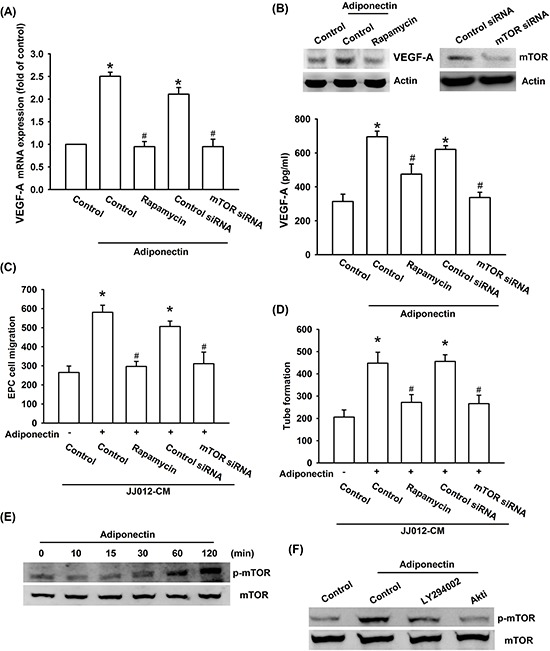
PI3K/Akt-dependent mTOR signaling pathway is activated in response to adiponectin treatment of human chondrosarcoma cells **A. & B.** Cells were pretreated with the rapamycin (30 nM) for 30 min or transfected with mTOR siRNA for 24 h followed by stimulation with adiponectin (10 ng/ml) for 24 h, and VEGF-A expression was examined by qPCR, ELISA and western blotting. **C. & D.** In addition, the medium was collected as CM and then applied to EPCs for 24 h. The capillary-like structures formation and cell migration in EPCs were examined by tube formation and Transwell assay. **E. & F.** Cells were incubated with adiponectin (10 ng/ml) for the indicated times, or pretreated with the ly294002 and Akt inhibitor for 30 min followed by stimulation with adiponectin for 60 min, and the mTOR phosphorylation was determined by western blotting. Results are expressed as the mean ± S.E.M. *, *p* < 0.05 compared with control; #, *p* < 0.05 compared with adiponectin-treated group.

Akt-dependent mTOR activation reportedly increases tumor angiogenesis [31]. We next examined whether Akt-dependent mTOR activation is involved in adiponectin-induced VEGF-A expression and angiogenesis. Our results showed that pretreatment of cells with mTOR inhibitor rapamycin or transfection of cells with mTOR siRNA abolished adiponectin-induced VEGF-A expression, EPC migration as well as tube formation (Figure 4A–4D). In addition, treatment of cells with adiponectin increased mTOR phosphorylation time-dependently (Figure 4E). Furthermore, pretreatment with ly294002 or Akt inhibitor markedly diminished adiponectin-induced mTOR phosphorylation (Figure 4F). Based on these results, it appears that the adiponectin acts through the PI3K, Akt and mTOR pathway to enhance VEGF-A expression in human chondrosarcoma cells.

### Involvement of HIF-1α in adiponectin-induced angiogenesis and VEGF-A expression

**Figure 5:**
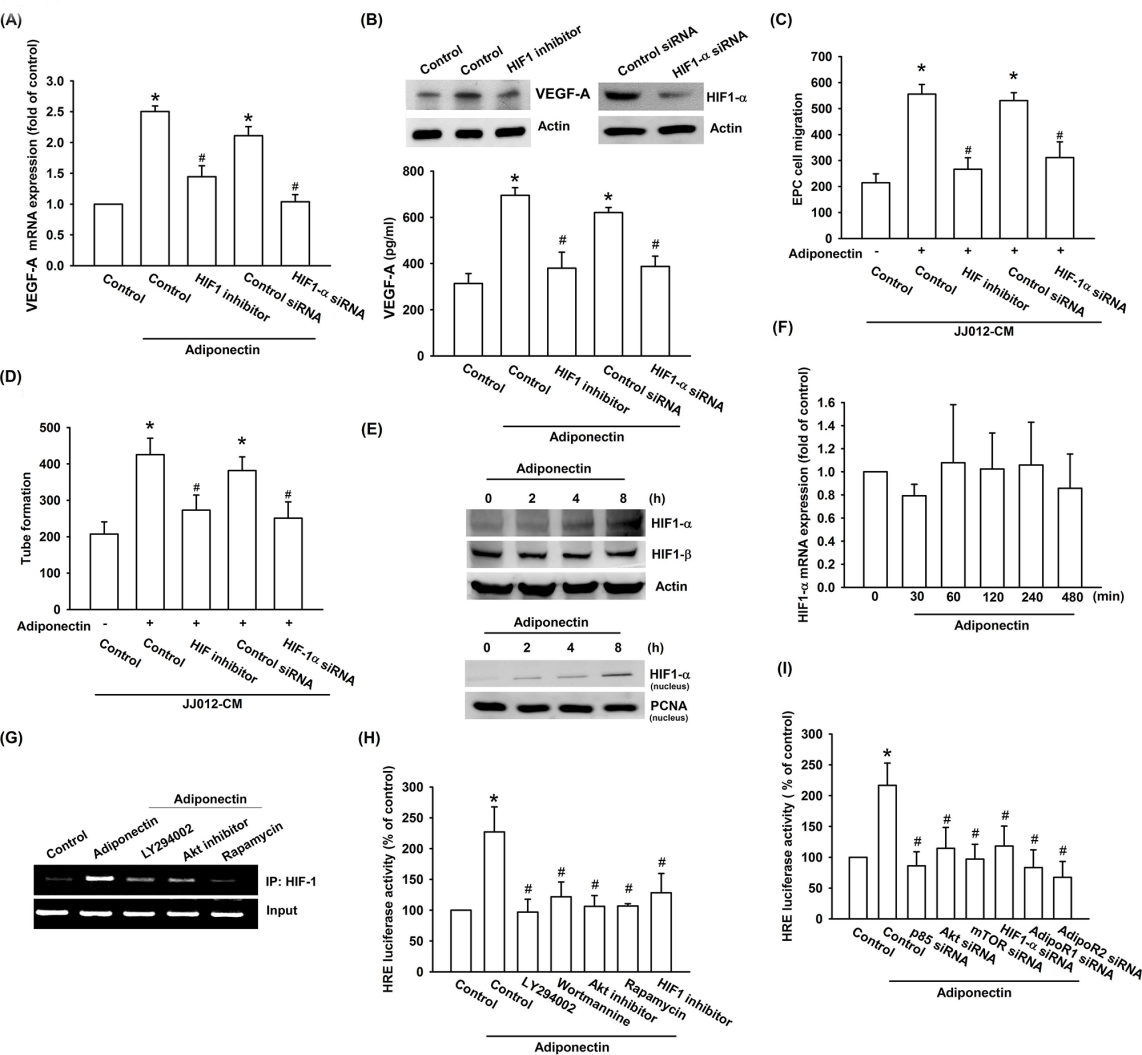
HIF-1α activation is involved in adiponectin-induced VEGF-A expression in human chondrosarcoma cells **A. & B.** Cells were pretreated with the HIF-1 inhibitor (10 μM) for 30 min or transfected with HIF-1α siRNA for 24 h followed by stimulation with adiponectin (10 ng/ml) for 24 h, and VEGF-A expression was examined by qPCR, ELISA and western blotting. **C. & D.** In addition, the medium was collected as CM and then applied to EPCs for 24 h. The capillary-like structures formation and cell migration in EPCs were examined by tube formation and Transwell assay. **E. & F.** Cells were incubated with adiponectin (10 ng/ml) for the indicated times and HIF-1α expression was determined by western blotting and qPCR. **G.** Cells were pretreated for 30 min with ly294002, wortmannin, Akt inhibitor, rapamycin, or HIF-1 inhibitor for 30 min followed by stimulation with adiponectin (10 ng/ml) for 120 min. The HIF-1α activation was examined by chromatin immunoprecipitation and HRE luciferase activity. **H. & I.** Cells were pretreated with ly294002, wortmannin, Akt inhibitor, rapamycin, or HIF-1 inhibitor for 30 min or transfected with siRNA of p85, Akt, mTOR, AdipoR1, AdipoR2, and HIF-1α before exposure to adiponectin. HRE luciferase activity was measured, and the results were normalized to β-galactosidase activity. Results are expressed as the mean ± S.E.M. *, *p* < 0.05 compared with control; #, *p* < 0.05 compared with adiponectin-treated group.

Hypoxia-inducible factor 1 (HIF-1), a pivotal transcription factor that is critical for VEGF-A expression in tumor angiogenesis [32]. We therefore sought to investigate whether HIF-1 activation was involved in adiponectin-induced VEGF-A expression in human chondrosarcoma cells. We found that pretreatment with HIF-1 inhibitor or transfection with HIF-1α siRNA both markedly antagonized adiponectin-induced VEGF-A expression (Figure 5A & 5B). Adiponectin-enhanced EPCs migration and tube formation were significantly suppressed by treatment with HIF-1 inhibitor and HIF-1α siRNA (Figure 5C & 5D). The results from Western blot indicated that adiponectin significantly increased protein level and translocation into nucleus of HIF-1α time-dependently (Figure 5E). However, adiponectin did not increase the mRNA level of HIF-1α using qPCR analysis (Figure 5F). We further analyzed the possible effect of adiponectin on HIF-1α protein synthesis, performed a time-course analysis of HIF-1α turnover in the presence of the protein synthesis inhibitor cycloheximide (CHX). According the Western blot result demonstrated that there was no significant change in HIF-1α protein half-life upon CHX treatment in the presence or absence of recombinant adiponectin (Supplementary Figure S2A), suggesting adiponectin enhanced the HIF-1α stability by increasing protein translation. In addition, transfection with AdipoR1 and R2 siRNA inhibited adiponectin-enhanced HIF-1α protein expression (Supplementary Figure S2B). Therefore, adiponectin increases the accumulation of HIF-1α through AdipoR1 and AdipoR2. We further explored whether PI3K, Akt, and mTOR signals were involved in adiponectin-induced HIF-1α activation in human chondrosarcoma cells. We performed ChIP assay to examine the DNA binding activity of HIF-1α in adiponectin-treated cells. As shown in Figure 5G, HIF-1α binding to the HRE element of the VEGF-A promoter occurred after adiponectin stimulation. The binding of HIF-1α to the HRE element by adiponectin was markedly attenuated by ly294002, Akt inhibitor, and rapamycin. Moreover, HIF-1α activation was also evaluated using HRE-luciferase assay. We found that adiponectin-induced HRE-luciferase activity was significantly reduced by pretreatment with ly294002, wortmannin, Akt inhibitor, rapamycin, and HIF-1 inhibitor or by transfection with AdipoR1, AdipoR2, p85, Akt, mTOR, and HIF-1α siRNA (Figure 5H & 5I). Based upon these finding, we suggest that AdipoR/PI3K/Akt/mTOR signaling pathway is involved in adiponectin-induced HIF-1α activation.

### Knockdown of adiponectin impairs angiogenesis *in vitro* and *in vivo*

**Figure 6:**
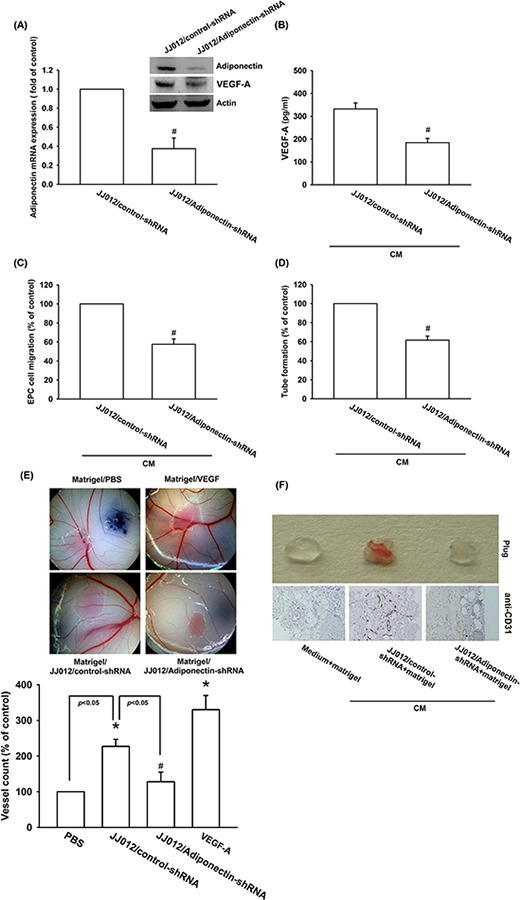
Knockdown adiponectin decreases VEGF-A expression *in vitro* and angiogenesis *in vivo* **A. & B.** The protein and mRNA levels of adiponectin and VEGF-A in JJ012/control-shRNA and JJ012/adiponectin-shRNA cells were measured with the western blotting, qPCR, and ELISA. **C.** EPCs cells were incubated CM from JJ012/control-shRNA or JJ012/adiponectin-shRNA for 24 h, the cell migration was examined by Transwell, **D.** and the tube formation was photographed under microscope. **E.** Chick embryos were incubated 4 day with serum-free medium, JJ012/control-shRNA CM, or JJ012/adiponectin-shRNA CM, and then resected, fixed and photographed with a stereomicroscope. **F.** Mice were injected subcutaneously with Matrigel mixed with serum-free medium, JJ012/control-shRNA CM, or JJ012/adiponectin-shRNA CM for 7 days. The plugs were excised from mice and photographed or stained with CD31. Results are expressed as the mean ± S.E.M. *, *p* < 0.05 compared with control; #, *p* < 0.05 compared with adiponectin-treated group.

To confirm the adiponectin increased VEGF-A-dependent angiogenesis in human chondrosarcoma cells, the adiponectin-shRNA expression cells was established. The expression of adiponectin and VEGF-A was reduced by adiponectin-shRNA in JJ012/adiponectin-shRNA cells (Figure 6A & 6B). We found that CM from JJ012/control-shRNA cells increased tube formation and migration of EPCs. Knockdown of adiponectin significantly suppressed CM-mediated EPCs migration and tube formation (Figure 6C & 6D). In addition, the effect of adiponectin on angiogenesis *in vivo* was evaluated by using the *in vivo* model of chick embryo CAM assay. CM from JJ012/control-shRNA cells increased angiogenesis in CAM was clearly observed. In contrast, adiponectin-shRNA markedly reduced angiogenesis in CAM (Figure 6E). We next performed the Matrigel implant assay in mice to further confirm adiponectin-increased angiogenic response *in vivo*. The results showed that Matrigel mixed with CM from JJ012/control-shRNA cells increased microvessel formation. Accordingly, CM from JJ012/adiponectin-shRNA cells significantly abolished neovascularization (Figure 6F). Knockdown of adiponectin also reduced microvessel formation in the Matrigel plugs by analyzing the CD31 (Figure 6F). Therefore, these results indicate that adiponectin plays an important role during chondrosarcoma-promoted angiogenesis *in vivo*.

### Knockdown adiponectin decreases tumor-associated angiogenesis *in vivo*

**Figure 7:**
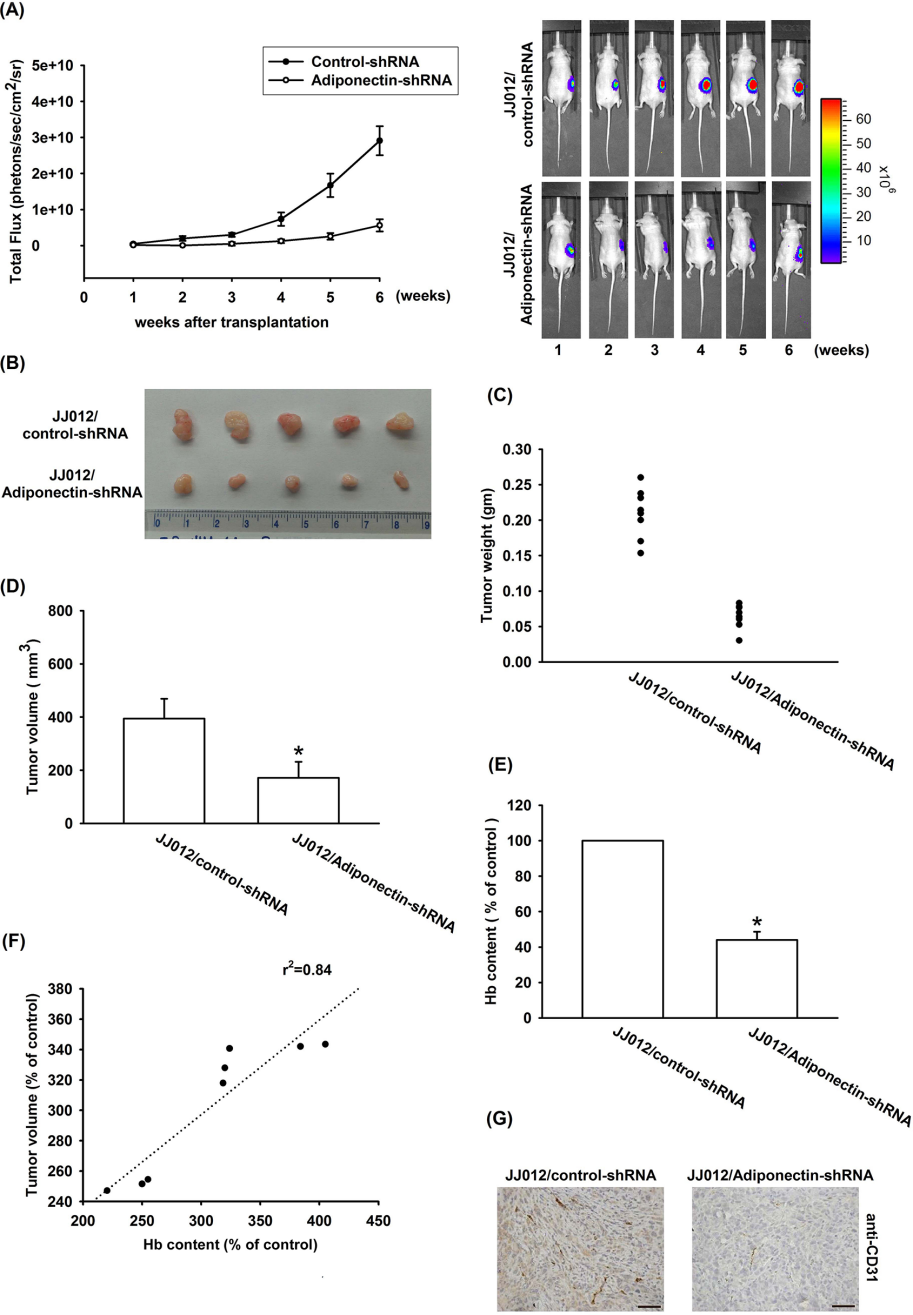
Knockdown adiponectin reduces tumor-associated angiogenesis *in vivo* **A–F.** JJ012/control-shRNA or JJ012/adiponectin-shRNA cells were mixed with Matrigel and injected into flank sites of mice then observed 5 weeks, and killed the mice resect of tumor. The tumors were photographed with a microscope, measured weight and volume, and quantified the hemoglobin levels. **G.** Immunohistochemistry of CD31 in tissues from chondrosarcoma cell xenografts. Results are expressed as the mean ± S.E.M. *, *p* < 0.05 compared with control.

In order to further investigate whether knockdown adiponectin was able to inhibit tumor-induced angiogenesis *in vivo*, a xenograft tumor-induced angiogenesis model was used. We respectively used two chondrosarcoma cell lines (JJ012/control-shRNA and JJ012/adiponectin-shRNA) mixed with Matrigel and injected into the right flanks of SCID mice. Observed 5 weeks by Xenogen IVIS system, and found that knockdown adiponectin reduced tumor growth in SCID mice (Figure 7A–7D). In addition, knockdown of adiponectin expression also reduced tumor-induced angiogenesis (Figure 7B). We quantified the level of angiogenesis by determining the hemoglobin content of the tumor and found that reducing adiponectin expression diminished chondrosarcoma-induced angiogenesis *in vivo* (Figure 7E & 7F). Finally, *ex vivo* analysis of tumors excised from mice showed significantly decreased CD31 expression in the JJ012/adiponectin-shRNA group (Figure 7G). Overall these results suggest that adiponectin promotes angiogenesis and tumor growth *in vivo*.

## DISCUSSION

Chondrosarcoma is estimated that it accounted for 3.6% of the annual incidence of all primary bone malignancies in the USA, after multiple myeloma and osteogenic sarcoma [33]. The distant metastatic potential of chondrosarcoma has already been reported [3, 4]. Patients who develop metastatic chondrosarcoma has poor prognosis. Tumor metastasis contains many processes, among of these processes the most important step is angiogenesis. Therefore, it is important to explore the potential target for preventing chondrosarcoma angiogenesis and metastasis. In the current study, we using immunohistochemistry analysis and found that the expression of adiponectin in chondrosarcoma patients correlated with tumor grade, and significantly higher than healthy cartilages. To best our knowledge, present study is the first time to correlate the expression of adiponectin in human chondrosarcoma patients. Adiponectin is abundant in human plasma, with concentrations ranging from 2 to 30 μg/ml. In the current study, we found that adiponectin 1 ng/ml increased VEGF-A expression in human chondrosarcoma cells. Therefore, the *in vivo* pathologic condition is more complicated than the *in vitro* culture system. Whether the increasing expression of adiponectin predicts for features of relapse-free and progression-free survival are needs further examination. On the other hand, chondrosarcoma CM-mediated tube formation and angiogenesis was abolished by adiponectin shRNA. In addition, adiponectin knockdown reduced angiogenesis and tumor growth *in vivo*. Our study suggests that adiponectin increased VEGF-A expression in human chondrosarcoma cells and subsequently promoted angiogenesis in EPCs. One of the mechanisms underlying adiponectin increased VEGF-A production through activation of AdipoR receptor, PI3K, Akt, mTOR, and HIF-1α pathways. Another chondrosarcoma cell line, SW1353 cells, was also observed that adiponectin is able to induce VEGF-A expression and enhance EPCs migration as well as tube formation via the same pathway (Supplementary Figure S3). These suggest that AdipoR/PI3K/Akt/mTOR/HIF-1α is a common pathway responsible for VEGF-A expression and angiogenesis in chondrosarcoma cells.

The adiponectin appears to play an important role not only in glucose and lipid metabolism but also in the development and progression of various types of cancers. In osteoblasts and breast cancer cells, adiponectin increased the *in vitro* motility, and it also augmented the expression of angiogenic growth factors [34, 35]. In current study, we indicated adiponectin promotes VEGF-A expression and angiogenesis but in prostate cancer cells the results instead [36], Gao Q et al. reported adiponectin suppresses growth of prostate cancer cells through inhibition of VEGF-A-mediated cancer neovascularization [36]. Since the mechanisms of action of adiponectin are not inconsistency and therefore a difficulty in ascertaining if the effect of adiponectin on cancer promotion is negative or positive.

Two adiponectin receptors, AdipoR1 and AdipoR2, that mediated the biological effects of adiponectin was identified [37]. AdipoR1 is a high-affinity receptor for globular adiponectin and a low-affinity receptor for the full-length ligand, whereas AdipoR2 is an intermediate-affinity receptor for both forms of adiponectin [37]. AdipoR1 is abundantly expressed in skeletal muscle, whereas AdipoR2 is predominantly expressed in the liver. Previous report documented that human chondrosarcoma expressed AdipoR1 and AdipoR2 receptor isoforms [26]. In addition, both AdipoR1 and AdipoR2 receptors involved in adiponectin-promoted metastasis in human chondrosarcoma [26]. In this study, we found that the AdipoR1 and AdipoR2 siRNA abolished adiponectin-induced VEGF-A expression. Furthermore, AdipoR1 and AdipoR2 siRNA also blocked adiponectin-enhanced tube formation in EPCs. These results indicated that adiponectin and AdipoR1/R2 interaction promoted VEGF-A expression in human chondrosarcoma cells.

HIF-1 is thought to play a major role in VEGF-A expression [38]. HIF-1α has been reported to activate VEGF-A expression by binding to the HRE site within the VEGF-A promoter [39]. Likewise, the activation of HIF-1α by adiponectin also resulted in an induction of VEGF-A transcription activity through the HRE site, although many other possible response elements, including activator protein-2, nuclear factor-κB, and simian virus 40 promoter factor 1, are located within the VEGF-A promoter. In current study, HIF-1 inhibitor or siRNA reduced adiponectin-induced VEGF-A expression and angiogenesis. Incubation of cells with adiponectin promoted HIF-1α but not HIF-1β expression time-dependently. However, adiponectin did not promote the mRNA expression of HIF-1α. It is apparent that adiponectin-induced VEGF-A expression is substantially mediated by the HRE. HIF-1α nuclear translocation is necessary for the transcriptional activation of HIF-1-regulated VEGF-A expression [40]. We subsequently demonstrated that adiponectin increased the binding of HIF-1α to the HRE element on VEGF-A promoter by ChIP assay. Using transient transfection with HRE-luciferase as an indicator of HIF-1α activity, we found that adiponectin also dramatically increased HRE-luciferase activity in chondrosarcoma cells. Therefore, HIF-1α is involved in adiponectin-induced VEGF-A expression.

HIF-1 has been reported to be regulated by a mTOR-dependent pathway [41, 42]. Numerous studies reported that PI3K/Akt/mTOR-dependent HIF-1α activation increases VEGF-A production [43, 44]. The present study found that the PI3K, Akt, and mTOR inhibitor or siRNA diminished adiponectin-induced VEGF-A expression. On the other hand, these inhibitors or siRNAs also antagonized the adiponectin-promoted the binding of HIF-1α to the HRE element on VEGF-A promoter and HRE-luciferase activity. Collectively, these data suggest that PI3K/Akt/mTOR/HIF-1α signaling pathway controls adiponectin/AdipoR axis-induced VEGF-A expression.

The rate-limiting step in metastasis, and a critical stage in cancer progression, is the acquisition of motility by a tumor cell. Lung metastasis is the major cause of mortality for patients with chondrosarcoma. In addition, angiogenesis is plays an important step in tumor metastasis. Thus, development of an anti-angiogenic and anti-metastatic therapy could conceivably be useful in these patients. Here we found that adiponectin induced VEGF-A expression and subsequently promoted angiogenesis and tumor growth in human chondrosarcoma cells through the activation of AdipoR1/R2 receptor, PI3K, Akt, mTOR, and HIF-1α signaling pathways. These findings may provide a better understanding of the mechanisms of angiogenesis and may lead to the development of effective therapies of chondrosarcoma.

## MATERIALS AND METHODS

### Materials

Anti-mouse and anti-rabbit horseradish peroxidase-conjugated IgG, rabbit polyclonal antibodies against AdipoR1, AdipoR2, β-actin, phosphorylated-p85 (p-p85), p85, phosphorylated-Akt (p-Akt), Akt, phosphorylated-mTOR (p-mTOR), and mTOR were purchased from Santa Cruz Biotechnology (Santa Cruz, CA, USA). HIF-1α, VEGF-A, and CD31 antibodies were purchased from Abcam (Cambridge, MA, USA). Recombinant human adiponectin and VEGF-A were purchased from R&D Systems (Minneapolis, MN, USA). pHRE-luciferase construct was provided from Dr. W.M. Fu (National Taiwan University, Taiwan). The pSV-β-galactosidase vector and luciferase assay kit were purchased from Promega (Madison, WI, USA). All other chemicals were purchased from Sigma-Aldrich (St. Louis, MO, USA).

### Cell culture

The human chondrosarcoma cell line (JJ012) was kindly provided by Dr. Sean P. Scully's laboratory (University of Miami School of Medicine, Miami, FL, USA) [45]. We used lenti-virus transfected adiponectin-shRNA or control-shRNA plasmids into JJ012 cells. After 48 h, replace with new medium. Subsequently, 10 μg/ml puromycin (Life Technologies) was used to select stable transfectants. Thereafter, the selection medium was replaced every 3 days. After 2 weeks of selection in puromycin, clones of resistant cells were isolated. The all JJ012 cells were cultured in Dulbecco's modified Eagle's medium (DMEM)/α-MEM supplemented with 10% FBS. These cells were maintained at 37°C in a humidified atmosphere of 5% CO_2_.

The human endothelial progenitor cells (EPCs) were from the study protocol was approved by the Institutional Review Board of Mackay Medical College, New Taipei City, Taiwan (reference number: P1000002), and all subjects gave informed written consent before enrollment in this study. After collecting peripheral blood (80 ml) from healthy donors, the peripheral blood mononuclear cells were fractionated from other blood components by centrifugation on Ficoll-Paque plus (Amersham Biosciences, Uppala, Sweden) according to the manufacturer's instructions. CD34-positive progenitor cells were obtained from the isolated peripheral blood mononuclear cells using CD34 MicroBead kit and MACS Cell Separation System (Miltenyi Biotec, Bergisch Gladbach, Germany). CD34-positive EPCs were maintained and propagated in MV2 complete medium consisting of MV2 basal medium and growth supplement (PromoCell, Heidelberg, Germany), supplied with 20% defined FBS (HyClone, Logan, UT). The cultures were seeded onto 1% gelatin-coated plastic ware and maintained at 37°C in a humidified atmosphere of 5% CO_2_ [8, 31].

### Western blot analysis

Cellular lysates were prepared as described previously [46]. Proteins (50 μg) were resolved on sodium dodecyl sulfate-polyacrylamide gel electrophoresis and transferred to Immobilon polyvinyl difluoride membranes. The blots were blocked with 4% nonfat milk for 1 h at room temperature and then probed with mouse anti-human antibodies against p-p85, p85, p-Akt, Akt, p-mTOR, mTOR, HIF-1α, VEGF-A, or β-actin (1:1000) for 1 h at room temperature. After 3 washes, the blots were subsequently incubated with a goat anti-mouse peroxidase-conjugated secondary antibody (1:1000) for 1 h at room temperature. The blots were visualized by enhanced chemiluminescence using Kodak X-OMAT LS film (Eastman Kodak, Rochester, NY).

### Quantitative real-time polymerase chain reaction

Total RNA was extracted from chondrosarcoma cells using a TRIzol kit (MDBio Inc., Taipei, Taiwan). The reverse transcription reaction was performed using 1 μg of total RNA that was reverse transcribed into cDNA using oligo(dT) primers [47]. Quantitative real-time PCR (qPCR) analysis was carried out using Taqman^®^ One-Step RT-PCR Master Mix (Applied Biosystems, CA). Two microliters of cDNA template was added to each 25-μl reaction with sequence-specific primers and Taqman^®^ probes. Sequences for all target gene primers and probes were purchased commercially. Glyceraldehyde 3-phosphate dehydrogenase (GAPDH) was used as an endogenous control to normalize expression data (Applied Biosystems). qPCR assays were carried out in triplicate on a StepOnePlus sequence detection system. The cycling conditions were as follows: initial 10-min polymerase activation at 95°C followed by 40 cycles at 95°C for 15 s and 60°C for 60 s. To calculate the cycle number at which the transcript was detected (CT), the threshold was set above the non-template control background and within the linear phase of target gene amplification.

### Transient transfection and reporter gene assay

Human chondrosarcoma cells were plated in 12-well dishes. DNA and Lipofectamine 2000 (LF2000; Invitrogen) were premixed for 20 min and then applied to the cells. Twenty-four hours after transfection, the cells were incubated with the indicated agents. Cell extracts were then prepared, luciferase and β-galactosidase activities were measured.

ON-TARGETplus siRNA of p85, Akt, mTOR, HIF-1α and control were purchased from Dharmacon Research (Lafayette, CO, USA). Transient transfection of siRNAs was carried out using DharmaFECT1 transfection reagent. siRNA (100 nM) was formulated with DharmaFECT1 transfection reagent according to the manufacturer's instruction.

### Preparation of conditioned medium (CM)

Human chondrosarcoma cell line (JJ012 cells) was plated in 6-well dishes, and cells were grown to confluence. Then culture media were changed serum-free DMEM/α-MEM medium. CM were collected 2 days after the change of media and stored at −20°C until use. In the series of experiments, JJ012 cells were pretreated for 30 min with inhibitors including ly294002, wortmannin, Akt inhibitor, rapamycin, HIF-1 inhibitor, or vehicle control (0.1% DMSO) and following by treated with adiponectin for 24 h to prevent signaling via the adiponectin.

### Migration assay

Migration activity was measured using a Transwell assay (Costar, NY; pore size, 8-μm). Approximately 1 × 10^4^ cells were added to the upper chamber in 200 μl of 10% defined FBS MV2 complete medium. The lower chamber in 300 μl of containing 150 μl 20% defined FBS MV2 complete medium and 150 μl CM. The plates were incubated for 24 h at 37°C in 5% CO_2_, and then cells were fixed in 3.7% formaldehyde solution for 15 min and stained with 0.05% crystal violet in PBS for 15 min. Cells on the upper side of the filters were removed with cotton-tipped swabs, and the filters were washed with PBS. On the other hand, cells on the underside of the filters were examined and counted under a microscope. Each clone was plated in triplicate for each experiment, and each experiment was repeated at least 3 times.

### ELISA assay

Human chondrosarcoma cells were cultured in 24-well culture plates. Cells were incubated in a humidified incubator at 37°C for 24 h. To examine the downstream signaling pathways involved in adiponectin treatment, cells were pretreated with various inhibitors for 30 min before add of adiponectin (10 ng/ml) administration. After incubation, the medium was removed and stored at −80°C until the assay was performed. VEGF-A in the medium was assayed using the VEGF-A enzyme immunoassay kits (Peprotech; Offenbach, Germany), according to the procedure described by the manufacturer.

### Chromatin immunoprecipitation assay

Chromatin immunoprecipitation analysis was performed as described previously [48]. DNA immunoprecipitated with an anti-HIF-1α Ab was purified and extracted with phenol-chloroform. The purified DNA pellet was subjected to PCR. PCR products were then resolved by 2% agarose gel electrophoresis and visualized with UV light. The primers 5′-CCTTTGGGTTTTGCCAGA-3′ and 5′-CCAAGTTTGTGGAGCTGA-3′ were utilized to amplify across the VEGF-A promoter region [49].

### Tube formation assay

Matrigel (BD Biosciences, Bedford, MA) was dissolved at 4°C overnight and 48-well plates were prepared with 100 μl Matrigel in each well after coating and incubating at 37°C overnight. EPCs (3 × 10^4^) in 200 μl cultured media which including 50% EGM-MV2 medium and 50% CM. After 6 h of incubation at 37°C, EPCs tube formation was assessed with a photomicroscope, followed which each well was photographed at × 200 magnification under a light microscope. Tube branches and total tube length were calculated using MacBiophotonics Image J software.

### Chick chorioallantoic membrane (CAM) assay

Fertilized chicken eggs were incubated at 38°C with 80% humidity. A small window was made in the shell on day 3 of chick embryo development under aseptic conditions. The window was resealed with adhesive tape and eggs were returned to the incubator until day 3 of chick embryo development. On day 7, CM from JJ012/control-shRNA or JJ012/adiponectin-shRNA cells (2 × 10^4^ cells) were mixed with Matrigel and deposited in the center of the chorioallantoic. At 11 days, *CAM* results were analyzed. Chorioallantoic membranes were collected for microscopy and photographic documentation. Angiogenesis was quantified by counting the number of blood vessel branch; at least 10 viable embryos were tested for each treatment. All animal works were done in accordance with a protocol approved by the China Medical University (Taichung, Taiwan) institutional animal care and use committees

### *In vivo* tumor xenograft study

Four weeks old male BALB/c-nu mice were used and assigned different treatments. For experimental cells growing exponentially, each implanted into 10 SCID mice by subcutaneous (SC) injection of 2 × 10^6^ cells (JJ012/control-shRNA or JJ012/adiponectin-shRNA) were resuspended in 200 μl of containing 50% serum-free DMEN/α-MEM and 50% Matrigel, then injected into the right flank. Observed 6 weeks by Xenogen IVIS system, then used CO_2_ to kill the mice. Then the tumor was removed and fixed in 10% formalin, and measured the tumor volume and weight. The hemoglobin content was examined by using the Drabkin's reagent kit.

### Matrigel plug assay

Matrigel plug angiogenesis assay was adapted from previously described assay [50]. Four-week male nude mice were assigned different treatments: serum-free medium, JJ012/control-shRNA CM, or JJ012/adiponectin-shRNA CM resuspended with Matrigel. Mice were subcutaneously injected with 300 μl Matrigel containing. After 7 days, Matrigel pellets were harvest, partly were fixed with 4% formalin, embedded in paraffin, and subsequently processed for immunohistochemistry stain for vessel marker CD31.

### Immunohistochemistry (IHC)

The human chondrosarcoma tissue array was purchased from Cybrdi (Rockville, MD, USA; 16 cases for healthy cartilage, 22 cases for grade I chondrosarcoma, 23 cases for grade II chondrosarcoma, and 21 cases for grade III chondrosarcoma); (Grade I chondrosarcoma grows relatively slowly, has cells whose histological appearance is quite similar to cells of healthy cartilage, and have much less aggressive invasive. Grades II and III are increasingly faster-growing cancers, with more varied and abnormal-looking cells, and are much more likely to infiltrate surrounding tissues). The tissues were placed on glass slides, rehydrated and incubated in 3% hydrogen peroxide to block the endogenous peroxidase activity. After trypsinization, sections were blocked by incubation in 3% bovine serum albumin in PBS. The primary antibody polyclonal rabbit anti-human adiponectin or VEGF-A were applied to the slides at a dilution of 1:200 and incubated at 4°C overnight. After being washed three times in PBST, the samples were treated with goat anti-rabbit IgG biotin-labeled secondary antibodies at a dilution of 1:50. Bound antibodies were detected with an ABC kit (Vector Laboratories). The slides were stained with chromogen diaminobenzidine, washed, counterstained with Delafield's hematoxylin, dehydrated, treated with xylene, and mounted. The intensity of staining was evaluated as 0, 1+, 2+, 3+, 4+, and 5+ for no staining, very weak staining, weak staining, moderate staining, strong staining, and very strong respectively under two independent and blinded observers. IHC score was determined as the sum of the intensity score.

### Statistical analysis

Data are presented as mean ± standard error of the mean. Statistical analysis of comparisons between 2 samples was performed using the Student's *t* test. Statistical comparisons of more than 2 groups were performed using one-way analysis of variance with Bonferroni's post-hoc test. In all cases, *p* < 0.05 was considered significant.

## SUPPLEMENTARY FIGURES


