# Supplementary material for: Adiponectin promotes VEGF-A-dependent angiogenesis in human chondrosarcoma through PI3K, Akt, mTOR, and HIF-α pathway
Source: Oncotarget. 2015 Oct 12;6(34):36746–61. doi: 10.18632/oncotarget.5479 (PMC4742208; doi:10.18632/oncotarget.5479)
Supplement: Supplementary file 1 [file oncotarget-06-36746-s001.pdf]

SUPPLEMENTARY FIGURES

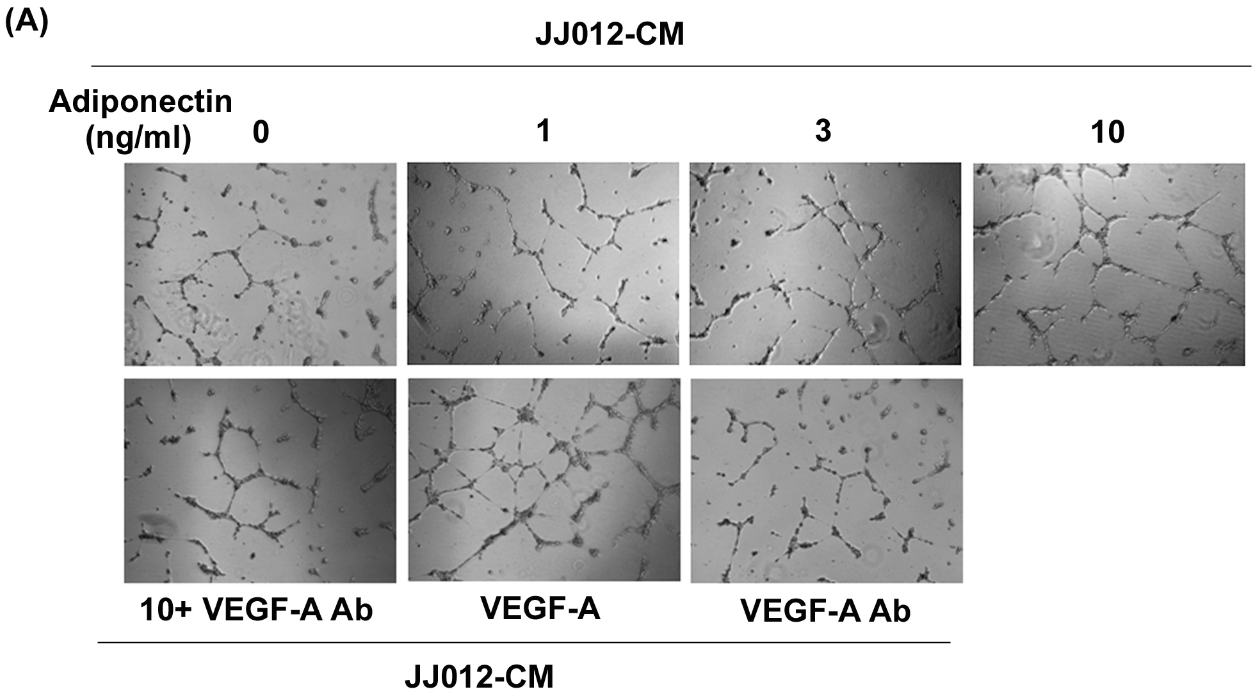

Supplementary Figure S1: Adiponectin promotes EPCs tube formation by raising VEGF-A expression in chondrosarcoma. The JJ012 cells were pre-treated for 30 min with VEGF-A antibody (5 μg/ml) followed by stimulation with adiponectin (10 ng/ml) or incubated with adiponectin (1–10 ng/ml) for 24 h. The medium was collected as CM and then applied to EPCs for 6 h. The capillary-like structure formation in EPCs was photographed and counted.

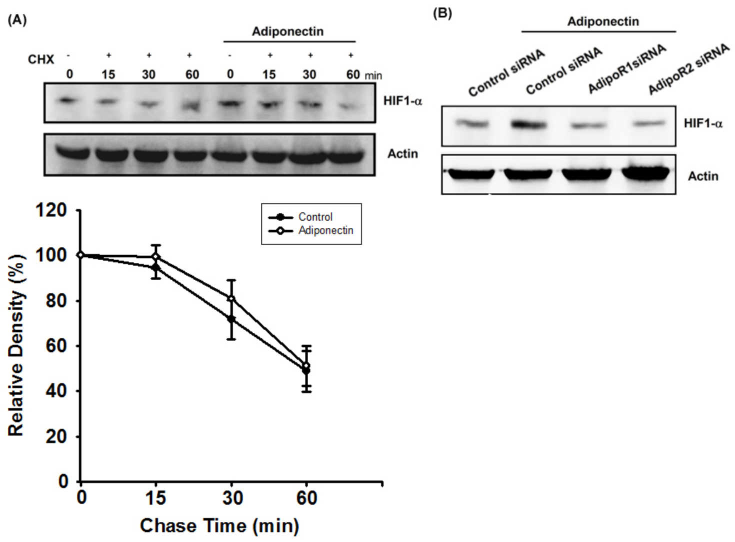

Supplementary Figure S2: Adiponectin induces HIF-1α expression through de novo protein synthesis. A. JJ012 cells were treated with adiponectin (10 ng/ml) for 8 h, then cycloheximide (CHX) 5 μM was added and incubated for 15–60 min. Total proteins were isolated, expression of HIF-1α was analyzed by Western blot assay (*top*). Quantification of HIF-1α protein expression by Western blot. (*bottom*). **B.** The JJ012 cells were transfected with AdipoR1 or AdipoR2 siRNA for 24 h followed by stimulation with adiponectin (10 ng/ml) for 8 h, and HIF-1α expression was examined by western blotting.

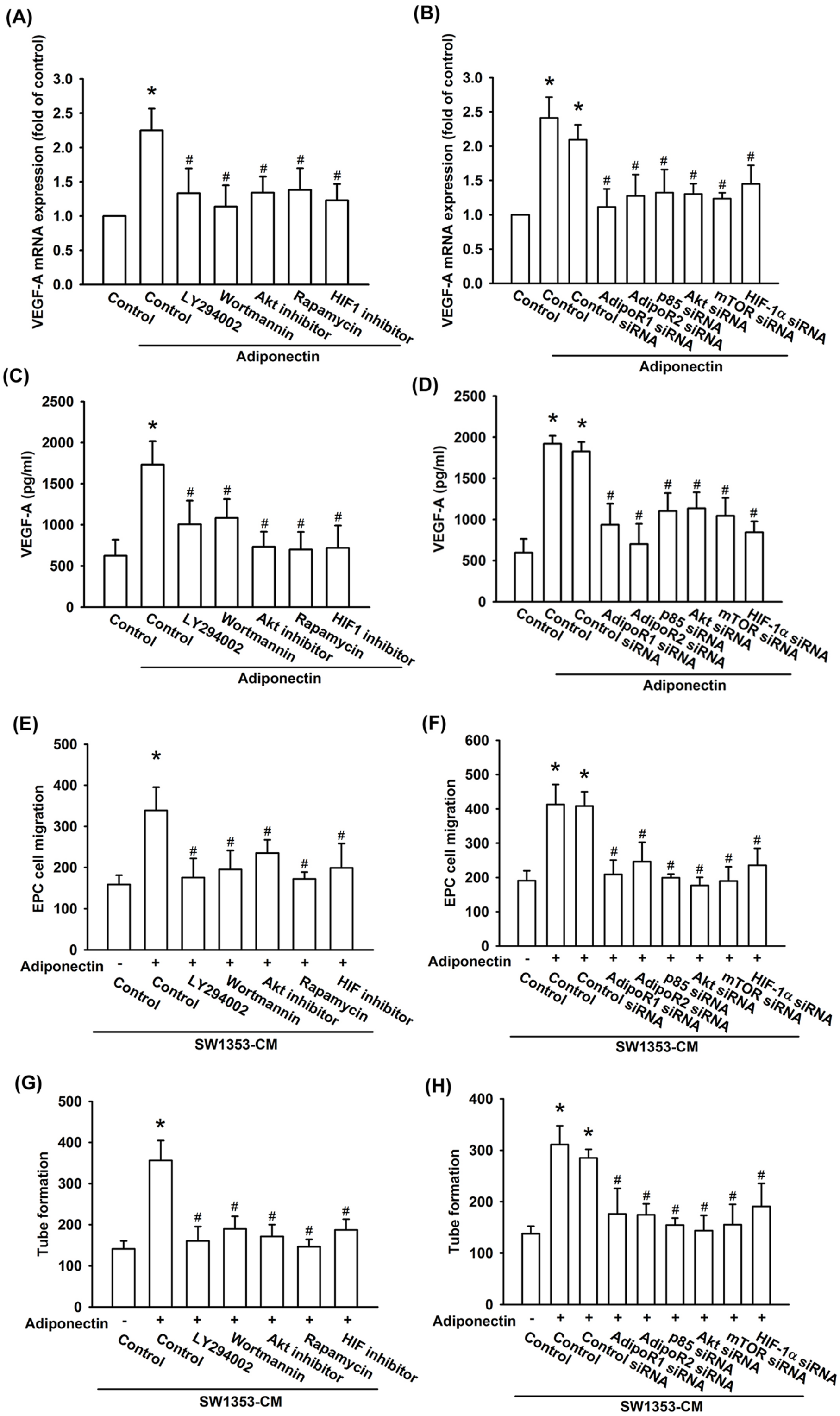

Supplementary Figure S3: Adiponectin promotes VEGF-A expression and angiogenesis through AdipoR, PI3K, Akt, mTOR, and HIF-1α signaling cascades in human chondrosarcoma cells. A–D. SW1353 cells were pretreated with the ly294002 (10 μM), wortmannin (150 nM), Akt inhibitor (10 μM), rapamycin (30 nM), or HIF-1 inhibitor (10 μM) for 30 min or transfected with AdipoR1, AdipoR2, p85, Akt, mTOR, or HIF-1α siRNA for 24 h followed by stimulation with adiponectin (10 ng/ml) for 24 h, and VEGF-A expression was examined by qPCR and ELISA. **E–H.** In addition, the medium was collected as CM and then applied to EPCs for 24 h. The capillary-like structures formation and cell migration in EPCs were examined by tube formation and Transwell assay. Results are expressed as the mean ± S.E.M. *, $p < 0.05$ compared with control; #, $p < 0.05$ compared with adiponectin-treated group.
